# Towards Better Precision Medicine: PacBio Single-Molecule Long Reads Resolve the Interpretation of HIV Drug Resistant Mutation Profiles at Explicit Quasispecies (Haplotype) Level

**DOI:** 10.4172/2153-0602.1000182

**Published:** 2015-11-08

**Authors:** Da Wei Huang, Castle Raley, Min Kang Jiang, Xin Zheng, Dun Liang, M Tauseef Rehman, Helene C. Highbarger, Xiaoli Jiao, Brad Sherman, Liang Ma, Xiaofeng Chen, Thomas Skelly, Jennifer Troyer, Robert Stephens, Tomozumi Imamichi, Alice Pau, Richard A Lempicki, Bao Tran, Dwight Nissley, H Clifford Lane, Robin L. Dewar

**Affiliations:** 1 Applied and Developmental Research Directorate, Leidos Biomedical Research, Inc., Frederick National Laboratory for Cancer Research, MD 21702, USA; 2 Advanced Biomedical Computing Center, Leidos Biomedical Research, Inc., Frederick National Laboratory for Cancer Research, MD 21702, USA; 3 Cancer Research Technology Program, Leidos Biomedical Research, Inc., Frederick National Laboratory for Cancer Research, MD 21702, USA; 4 Division of Clinical Research, National Institute of Allergy & Infectious Diseases, USA; 5 Critical Care Medicine Department, National Institutes of Health, Bethesda, MD, 20892, USA; 6 National Human Genome Research Institute, National Institutes of Health, Rockville, MD, 20852, USA; 7 National Cancer Institute, National Institutes of Health, Bethesda, MD, 20892, USA

**Keywords:** PacBio, Tag-sequence, HIV-1 drug resistance mutation, Next generation sequencing, Third generation sequencing, Quasispecies, Haplotype, Linkage, Single nucleotide variant

## Abstract

Development of HIV-1 drug resistance mutations (HDRMs) is one of the major reasons for the clinical failure of antiretroviral therapy. Treatment success rates can be improved by applying personalized anti-HIV regimens based on a patient’s HDRM profile. However, the sensitivity and specificity of the HDRM profile is limited by the methods used for detection. Sanger-based sequencing technology has traditionally been used for determining HDRM profiles at the single nucleotide variant (SNV) level, but with a sensitivity of only ≥ 20% in the HIV population of a patient. Next Generation Sequencing (NGS) technologies offer greater detection sensitivity (~ 1%) and larger scope (hundreds of samples per run). However, NGS technologies produce reads that are too short to enable the detection of the physical linkages of individual SNVs across the haplotype of each HIV strain present. In this article, we demonstrate that the single-molecule long reads generated using the Third Generation Sequencer (TGS), PacBio RS II, along with the appropriate bioinformatics analysis method, can resolve the HDRM profile at a more advanced quasispecies level. The case studies on patients’ HIV samples showed that the quasispecies view produced using the PacBio method offered greater detection sensitivity and was more comprehensive for understanding HDRM situations, which is complement to both Sanger and NGS technologies. In conclusion, the PacBio method, providing a promising new quasispecies level of HDRM profiling, may effect an important change in the field of HIV drug resistance research.

## Introduction

HIV strains are dynamic in the host during infection, with distinct characteristics of high turnover and mutation rates. Many mutations can arise from one HIV generation to the next, resulting in genetic diversity of the HIV population, termed “quasispecies” [1–4]. Under the pressure of certain antiretroviral treatments, some HIV quasispecies with drug resistance mutations (HDRMs) are preferentially selected and propagate. Clinically, the drug resistance quasispecies can become the dominant strain in the patient, thereafter making the treatment ineffective [4]. Different HDRM profiles are resistant to different types of antiretroviral drugs. For example, mutation of D30N in HIV protease usually resists NFV, but not for other anti-protease drugs. These types of correlations have been documented and recorded in a large drug resistance knowledge database [5]. Monitoring the HDRM profile of patients provides not only important guidance to formulate different combinations of drugs in a personalized/stratified medicine practice by utilizing appropriate sensitive drugs and avoiding insensitive drugs, but also to accumulate new knowledge regarding the correlation between HDRM profiles and the response to treatment regimens.

**Figure 1:**
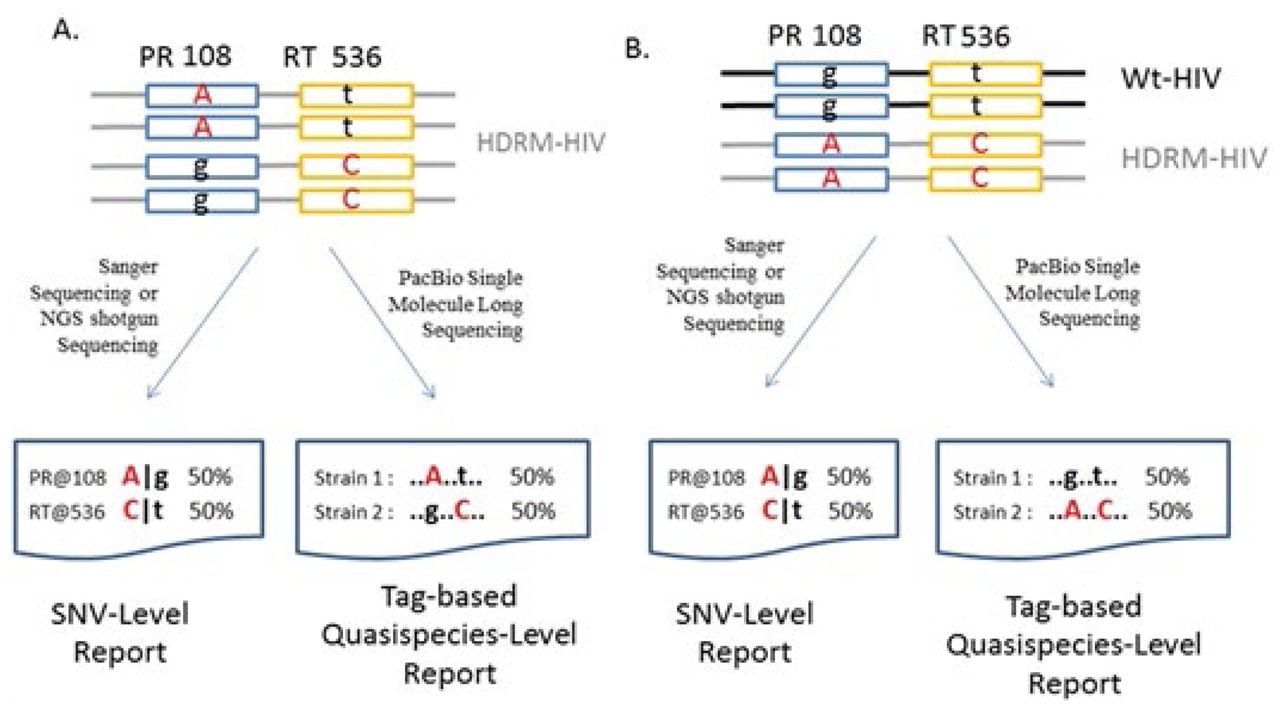
Illustration of Single Nucleotide Variant (SNV) and Quasispecies-level Analysis. The upper-case letter in red color is denoted as a mutation. The small-case letter in black is denoted as a wildtype (WT) nucleotide **A.** HIV population has two sub populations (quasispecies). The SNV-level report lists two individual mutations in the HIV population at position 108 of PR and position 536 of RT separately. In contrast, the quasispecies-level report lists two tag-sequences (representing the corresponding HIV quasispecies) of “..A..t…” and “..g..C..” by linking the two nucleotide calls at the position of interest. B. HIV population has different quasispecies composition with a distinct sub population of WT HIV. The SNV-level report gives a result identical to that of A, even though the two HIV populations have different compositions. The SNV-level report does not distinguish the different situations due to lack of haplotype information. In contrast, the quasispecies-level report reflects the difference in precision.

Our traditional approach of detecting HDRMs is the Sanger-based sequencing system (such as TruGene^®^) that amplifies and sequences the 1.4-kb POL region (the HIV genomic region encoding the viral enzymes protease, reverse transcriptase, and integrase) of the HIV virion population in a patient sample. As a population-based sequencing method, TruGene^®^ is able to reliably detect single nucleotide variants (SNVs) at ≥ 20% in the HIV population of a patient [6]. Next Generation Sequencing (NGS) technologies, such as 454 and Illumina, offer greater sensitivity (~ 1%) and higher throughput (hundreds of samples per run) compared to the TruGene Sequencing System [7–10]. Regardless of these promising improvements from NGS, both TruGene^®^ and NGS systems have a common weakness, that is, their detection capabilities are limited to the individual single nucleotide variant (SNV) level instead of the quasispecies level necessary for complete HDRM profiling (Figure 1A). Because the shorter reads lack the linkage information among the individual mutations, it is very difficult to sort the short reads of NGS in a big mixture and assemble them into each corresponding quasispecies [9]. The SNV view of HDRM profiling is the dominant approach employed in HIV drug resistance research and clinical application. In the current paradigm, a list of individual drug resistance mutations correlating to the drug resistance phenotype is reported, regardless of the potential relationship of the multiple mutations as a co-functional unit (Figure 1B). The multiple mutations within each HIV genome interact cooperatively on a functional level and make an integrative contribution to the final characteristics of the HIV quasispecies. Thus, a quasispecies HDRM view is a way to more precisely evaluate the drug resistance situation. The quasispecies profiling model more closely represents the nature of how HIV functions at the quasispecies level, rather than the individual mutation level, to effect drug resistance. Unfortunately, both Sanger sequencing and NGS shotgun sequencing are population-based detection (as opposed to single molecule/genome detection) so that their resolution is difficult at haplotype/quasispecies level.

To obtain quasispecies-level HDRM profiling, the technique of Single Genome Analysis (SGA) can be used. SGA is accomplished by diluting the HIV population from an individual into single copies in each well of a 96-well plate, and then amplifying and sequencing the single HIV copies separately [11]. This approach bypasses the complications of population-based sequencing by diluting the diversity of HIV genomes into a singleton so that the resolution is at quasispecies level. In this way, any observed mutations derived from the same well of the 96-well plate are known to stem from one single quasispecies. However, this method is extremely labor-intensive, time-consuming and low-throughput. These characteristics of SGA render it impractical either as a routine assay in the lab or for a large-scope study.

Moreover, building re-construction algorithms to sort the population-level shotgun-read mixture generated by NGS into small numbers of contigs at the quasispecies-level have been explored. Even though re-constructing the haplotype contigs from a shotgun-read mixture were reported with successes in some cases [12,13], the reliability of the re-construction algorithms in real-life studies is still questionable [9,14,15]. In our practice, the re-construction algorithms repeatedly generated false positives, e.g. chimerical contigs. Thus, to date, the SNV level of HDRM is still the dominant way in the drug resistant field.

The newer PacBio RS II platform from Pacific Biosciences provides a new potential to study HDRM profiling due to its distinct characteristics of very long (up to 40 kb), single molecule reads [16–18]. The PacBio long reads allow each targeted HIV molecule (usually a bulk PCR product of HIV population) to be continuously sequenced at full-length in one contiguous pass so that the linkage information between every individual mutation on the same HIV molecule is well characterized. The ability of the high-throughput PacBio technology makes feasible the large-scale study of HIV drug resistance at the important quasispecies-level, where other systems have had difficulty, as previously discussed (Figure 1A). However, since the PacBio long reads have higher error rate, special precautions during data analysis must be applied using an appropriate bioinformatics method in order to fully utilize the advantages resulting from having long reads to characterize full-length, contiguous regions of individual HIV molecules [17].

In this report, we first made benchmark samples by admixing two slightly different HIV strains in different ratios to serve as HIV quasispecies positive controls. The benchmark samples were thereafter used to evaluate the sensitivity of quasispecies-level detection using PacBio single-molecule, real-time sequencing. Finally, the PacBio system was applied to two case studies of clinical HIV samples at quasispecies-level HDRM detection.

## Materials and Method

### Generating benchmark samples

RNA was extracted from HIV pLN4-3 strains by Qiagen RNA kit. RNA of HIV BH10 was obtained from TruGene commercial kit. TruGene proprietary RT-PCR kit was used to amplify 1.4-kb POL region of HIV BH10 strain and pLN4-3 strain respectively. The DNA products derived from the two RT-PCR reactions were purified and quantitated. Thereafter, serial 2-fold admixture samples of the two DNA products were made at different ratios from 20%, 10%, 5%, and so on until 0.125% (Table 1).

### Preparation of clinical samples

HIV RNA was extracted by the Qiagen RNA extraction kit. TruGene proprietary RT-PCR kit was used to amplify the 1.4-kb POL region. The PCR products are directly submitted to TruGene sequencing. The PCR products were also purified by Qiagene DNA purification column kit. The purified DNA products are ready for MiSeq or PacBio deep sequencing.

### TruGene sequencing and data analysis

The PCR product was subjected to the TruGene sequencer according to the manufacture’s protocol. The output sequences in fasta format were compared to the wild type reference sequence of HIV LAV1 for SNV analysis by the TruGene built-in software package. A list of SNVs was reported as the final results [19].

### Illumina MiSeq deep sequencing and data analysis

One ng of DNA sample was used to construct MiSeq library by the Illumina Nextera kit. Standard MiSeq flow cell and 2×150 cycle MiSeq sequencing kit v2 were used for the deep sequencing. The output sequencing data was converted and demutiplexed into fastq format files by the MiSeq Sequence Analyzer.

The SNV procedure was done in following order:

1. BWA was used to aligned the reads to the wild type reference sequence of HIV LAV1.
2. SAM tools were used to generate pileup files based on BWA’s alignment file.
3. VarScan was used to make variant calls from the pileup file. A list of SNV with corresponding frequencies was reported as the final results.

### PacBio deep sequencing and tag-based quasispecies data analysis

One μg of the DNA product was used to construct the PacBio DNA library using the PacBio standard 2 kb template prep protocol. The samples were sequenced on the PacBio RS platform on a single SMRT Cell per sample. C2 Polymerase was used for the sequencing reaction and ninety-minute movie windows were used for signal detection. After raw sequence data were generated, the base calling and CCS read generation were done using version 1.3.0 of PacBio’s instrument control and SMRT Analysis software with default parameters. The output sequences are in fastq format.

**Figure 2:**
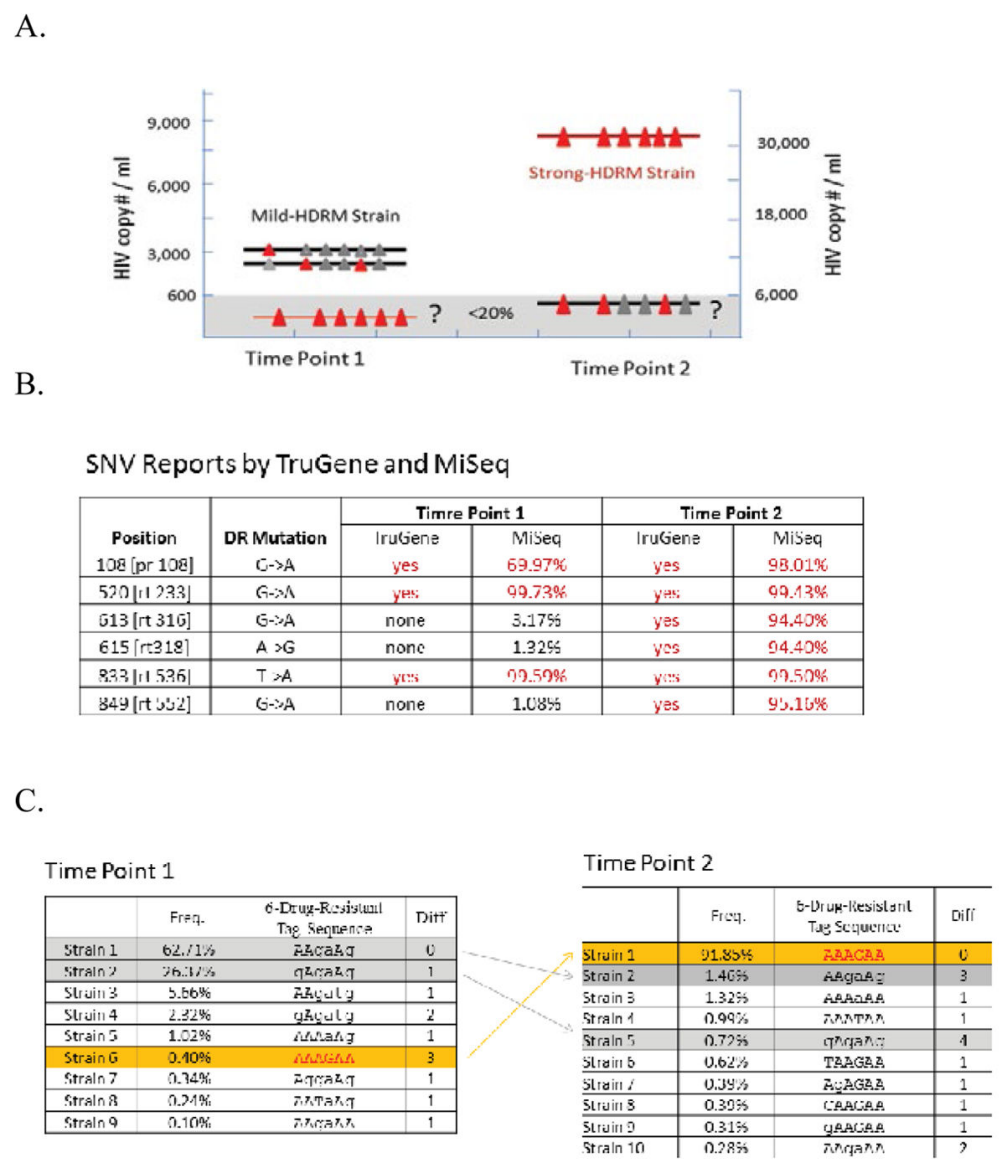
Case study 1 shows the dynamics of HIV drug resistant mutation (HDRM) across two time points A. Plot illustration of hypothesis of the potential HDRM changes across two time points. The triangle in red denotes drug resistance mutation at the position, and the one in black is wildtype. The gray area means the <20% frequency area that is below the detection sensitivity of traditional Sanger based TruGene sequencing. The question is whether the HDRM seen existed in the time point 1. B. The SNV-analysis results are derived from TruGene and MiSeq. MiSeq is able to detect 3 additional mutations (613, 615 and 846) with lower frequencies around ~ 1% in the time point 1. The HDRM seem to exist in very low frequencies, but without confidence, because the frequencies are at the boundary of MiSeq detection sensitivity. **C**. The quasispecies-level analysis of the HIV population was done based on PacBio datasets for the two time points. The positions of 6 drug resistance mutations are selected into the tag panel. The tag-sequences show that HDRM clearly exist at very low frequency in the time point 1, and becomes the dominant quasispecies in the time point 2. Of note, the table lists all potential quasispecies. The tag-sequences with difference value <2 and frequency <0.4% should be considered as noise.

**Figure 3:**
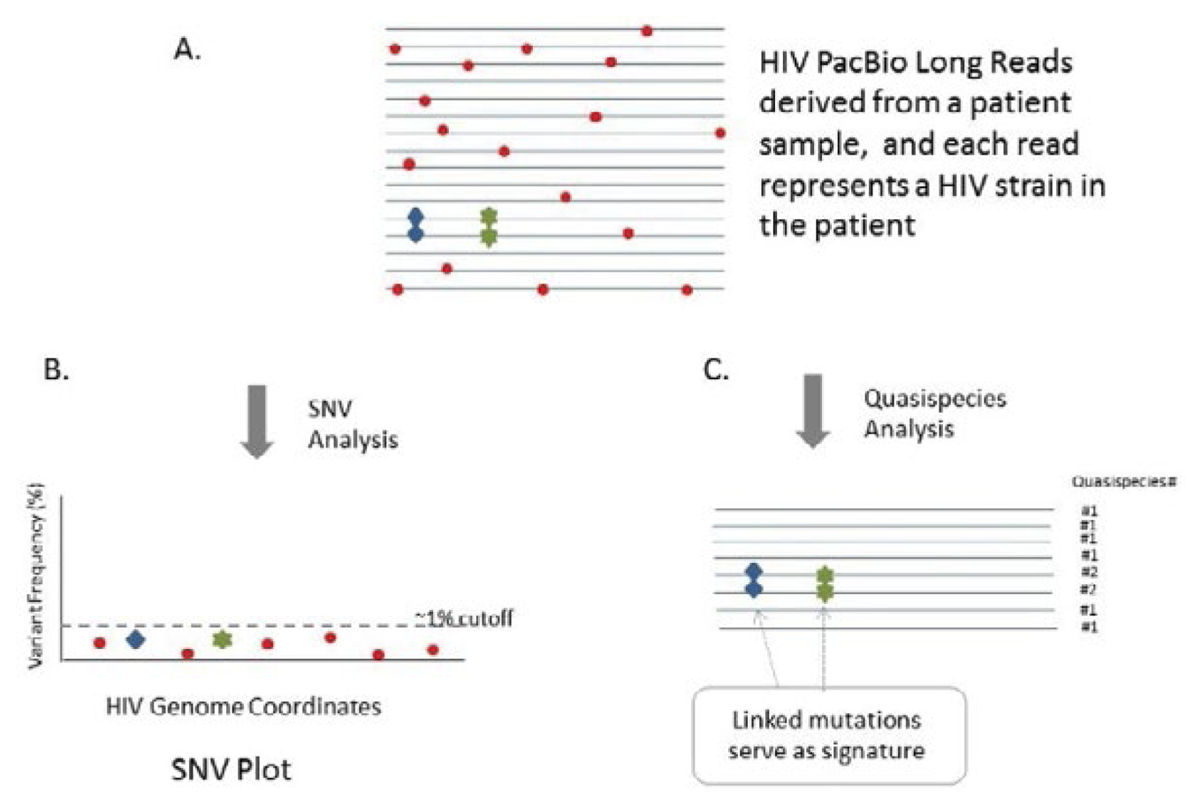
The concepts of signal-noise separations of SNV-analysis vs. quasispecies-analysis. The red dots are denoted as higher sequencing errors on the PacBio long reads. The blue and green marks represent two true mutations A. The PacBio reads contain the mixed signals of the true mutations (in blue and green) and many errors (in red). Of note, the errors are in random distribution. B. Individual SNV-analysis is to measure the frequency of the variant (comparing to the wildtype reference sequence) for each given position in a one-by-one independent way. The frequencies of each position can be combined and presented in a SNV plot. The background noise (dots in red) is at the level of ~ 1% for both MiSeq and PacBio. If an individual true mutation (marks in blue or green) is at the noise area, it is difficult to distinguish it from the background noise. **C.** The PacBio errors are randomly distributed, so that it is difficult to form a co-occurrence of two errors on the same read by chance. In contrast, the co-occurrence of two true mutations (in blue and green) with a relatively high frequency could be easily distinguished from noise signals. The co-occurrence pattern of quasispecies is the most important foundation for the tag-based quasispecies analysis.

The tag-based quasispecies analysis procedure was done in the following order (Figure 1):

1. The SNV procedure was done in a similar way as the MiSeq procedure except using PacBio’s Blasr for the mapping step instead of BWA. PacBio’s Blasr was found to be more tolerant of the high error rate of PacBio reads [20].
2. Tag positions were selected for the tag panel based on a combination of prior drug-resistance mutation knowledge and the results of the SNV analysis.
3. An in-house Perl script using BIO::BAM library was developed to query a tag sequence for each read, and then to create a tag-sequence by linking the nucleotide of each tag position in order. At this point, each tag sequence represents a quasispecies (Figure 1).
4. The script further clustered the tag- sequences to calculate their frequencies in the population. Any tag-sequence groups with more than 10 tag-sequences were selected for further evaluation. In addition, a “difference” value was calculated for each tag-sequence by comparing it to the dominant (most frequent tag-sequence) quasispecies. For example, the first dominant drug resistant tag-sequence of “AAAGAA” has 3 nucleotide differences comparing to the second drug resistant tag-sequence of “AAgaAg” (Figure 2). Tag-sequences with a difference value ≥ 2 were considered more trustworthy unless they occurred at a frequency greater than 0.4% (Figure 3, 2B) in the final consideration.

## Results

### HIV benchmark studies: PacBio offers a clear view of HIV quasispecies diversity

**Figure 4:**
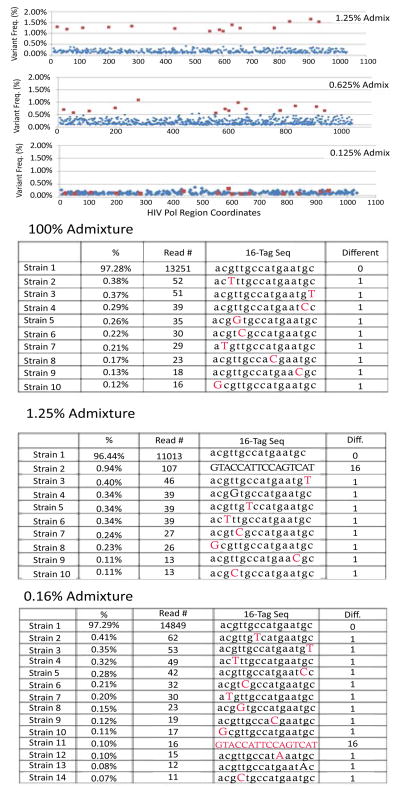
Benchmark study shows PacBio’s quasispecies-analysis is more comprehensive than MiSeq’s SNV-analysis A. The SNV plots derived from MiSeq SNV analysis on benchmark admixture samples of 1.25%, 0.625% and 0.125%. The background noise (in blue) is in general low level (<1%). The expected 16 SNVs (in red) are well separated from background for the 1.25% benchmark sample. The separation of signal (in red) and noise (in blue) start to be blurred on the 0.625% benchmark sample, and finally lost on the 0.125% benchmark sample. B. The tag sequences derived from PacBio’s quasispecies analysis on the same benchmark samples using the 16 SNVs at a tag panel. After the positions of 16 expected SNVs were selected into tag panel (signature) to construct artificial tag-sequence representing, the quasispecies profile is simply presented by the tag-sequences. For the tag-sequence, the lower case letter in black denotes wildtype (wt) nucleotide of the position. The upper case letter in red denotes a mutation of the position. The column of ‘difference’ values is to measure the number of mutations co-occurring on the same quasispecies by comparing it to the most frequent tag-sequence. According to the co-occurrence concept illustrated, the tag sequences with the difference number <2 and frequency <0.4% should be considered as noise or unconfident records. With these criteria, the minor quasispecies can easily stand out in the 0.165% admixture sample (Figure 3).

The HIV benchmark samples, artificially formed by admixing two slightly different HIV strains at different ratios (Table 1), serve as positive controls to evaluate the minor quasispecies detection ability of PacBio. Average sequencing yield produced approximately 20,000 qualified Circular Consensus Sequence (CCS) reads in full length (Table 1). After the tag-based analysis method was used to analyze the CCS reads at the quasispecies level, the CCS reads were clustered into a smaller number of tag-sequence groups. Each of the tag-sequence groups, a unique combination of artificially linked nucleotides extracted from tag positions of CCS reads, represents a quasispecies in the HIV population (Figure 1 and 4); also see method for details. The quasispecies-level view is able to discover the minor quasispecies down to 0.1% prevalence in the benchmark samples (Figure 4B).

In a cross-platform comparison with Illumina, the same benchmark samples were also sequenced by MiSeq. We conducted an SNV analysis with a similar data processing workflow used by other reported studies [9,10,21,22]. The ~ 1% sensitivity is confirmed with very stable performance at the individual SNV level of analysis (Figure 4A). However, Illumina SNV analysis is unable to reach to 0.1% detection sensitivity. In addition, the re-construction algorithms were also tested on the benchmark samples by a set of software packages including Newbler, Celera Assembler, CLC Bio, MIRA, MINIMUS, Quiver, Vicuna, and PredictHaplo [21]. The results contain high level of chimeric contigs of artifacts (data not shown) consistent with the discussions in other reports [12,13].

The PacBio single molecule long reads obviously offer a unique sequencing ability of maintaining the linkage information of every individual mutations on the same HIV quasispecies strain, that shorter reads from other NGS platforms (such as 454 and Illumina) cannot do. In order to analyze error-prone sequences, a tagging method was used to identify high confidence SNVs and then tag each quazispecies for the presence or absence of each tag SNV. In this way, HIV haplotypes were identified in the benchmark study and exampled clinical studies. The important bioinformatics strategy described in the paper is the tag-based approach, which makes the data analysis to focus on most important spots rather than being distracted by the higher background noises. More specifically speaking, the errors among the single-pass subreads are randomly distributed during sequencing such that the chance of an error recurring at the same tag positions on every subread of each single-molecule sequencing template is extremely low [17]. The promising detection sensitivity of quasispecies-level analysis comes from the co-occurrence pattern of multiple mutations on the same read, which is very difficult to form by random sequencing errors (Figure 3). For example, if mutation 1 has a 5% random chance of being miscalled as a sequencing error, and mutation 2 also has a 5% chance, then the chance of both mutation 1 and mutation 2 being miscalled on the same strain, denoted as the co-occurrence rate, is 0.25% (5% × 5%). Thus, the tag-based quasispecies-level analysis produces much more sensitive detection than the individual-SNV analysis because it takes into consideration the error rate of neighboring mutations on the same read (Figure 3). While the tag-sequence containing randomly distributed errors could not form a strong tag-sequence when present at very low frequencies, errors can be easily filtered out by the criteria such as minimum number of reads supporting the tag-sequence and minimum number of co-variants among the tags (Figure 3).

Moreover, defining a meaningful tag panel is an essential foundation of the quasispecies analysis when using the tag-based approach. We found that a tag number greater than three may give better results, because smaller number of tags (e.g. <= 2) do not provide an advantage over SNV level analysis (Figure 3). In another words, smaller number of tags (e.g. ≤ 2) is closer to SNV level analysis (Figure 3). PacBio being as a complement to the TruGene or Illumina platforms, the tag panel can be selected based on systematic selection from their SNV analysis results (e.g. all SNVs with frequency greater than 1%). For example, sixteen tag positions were selected for use in the tag panel for this benchmark study based on a clear statistical significance reported by the SNV analysis (Figure 4A) of Illumina. In a drug resistance study, the tag panel can also be built with the prior-known drug resistance mutation positions in the HIV drug resistance database.

#### Case study 1: The PacBio quasispecies-level analysis is able to detect minor drug resistant quasispecies

An HIV-infected patient had a sudden increase of HIV plasma viral load within one month from ~ 3,000 copies/ml at time point 1 to ~ 30,000 copies/ml at time point 2 (Figure 2A). The TruGene standard drug resistance detection showed a profile of three mutations in the protease (PR) and reverse transcriptase (RT) regions at time point 1, with a drug-sensitive interpretation. One month later, the profile contained three additional mutations in the RT region and there was a dramatic increase in viral load to ~ 30,000 copies/ml at time point 2 and the loss of sensitivity to two classes of antiretroviral drugs (ARV) (Figure 2A). An HDRM-development question is raised: how did the HIV drug resistance profile change from a drug-sensitive profile to a drug-resistant profile within just one month?

There are two possible scenarios as illustrated in (Figure 2A):

1. Drug treatment induces hypermutation and thus the introduction of new drug-resistance mutations.
2. The drug-resistant quasispecies already existed as a minor species in the HIV population at time point 1. Under the drug treatment pressure, they are quickly selected for and become dominant by time point 2. If so, early treatment of the low frequency drug resistant quasispecies at time point 1 might prevent their outgrowth and the resultant increase in viral load [23,24] (Figure 2A).

The standard TruGene system was able to report the HDRM at the SNV-level, as described earlier, for time points 1 and 2 (Figure 2B). There was no indication that the three additional mutations existed in low frequencies in time point 1, (Figure 2A and 2B). Therefore, the HDRM-development question raised earlier cannot be answered by the TruGene platform. However, given the sensitivity cutoff of TruGene of 20%, it is still possible that these mutations were present at the earlier time point at a lower frequency.

To take advantage of the higher detection sensitivity of NGS, the same HIV samples from the two time points were also sequenced by the Illumina MiSeq. MiSeq deep-sequencing yields much more sensitive SNV detection than that produced using TruGene [10]. MiSeq’s SNV report was able to detect the three additional mutations of the second HDRM profile at frequencies of 3.17%, 1.32%, and 1.08% in the time point 1 sample (Figure 2B), which are frequencies at the MiSeq’s lower detection limit, as suggested in the benchmark section (Figure 4A). At this point, MiSeq’s SNV analysis suggests that the drug-resistant HDRM might exist at lower frequencies at time point 1. But without further linkage information, it is not clear if the three mutations are on the same HIV strain or scattered among different strains.

The same HIV samples from two time points were thereafter sequenced by the PacBio system. PacBio was able to generate approximately 20,000 qualified CCS reads in full length or near full length for each sample. The positions of six previously known drug resistance mutations were selected into the tag panel for tag-based quasispecies analysis based on the relevancy of the SNVs at locations known to convey drug resistance. The tag-based quasispecies analysis was able to build clear tag-sequences representing the HIV quasispecies. The results clearly showed a minority variant with the drug-resistant profile was present at a very low frequency level (0.3%) in time point 1, and this was identical to the dominant variant at time point 2 (Figure 2C). At this point, PacBio quasispecies-level analysis provided the clearest dynamic view of HDRM profiles across the two time points. The PacBio results answered the earlier HDRM development question indicating the patient situation is best described by the second scenario, i.e. the drug resistant strain existed at a low frequency in early time point 1, only to become dominant following drug-resistance selection. This explains why the drug-resistant profile was able to arise so quickly within one month. The high sensitivity and clear quasispecies-level analysis enabled by long reads from PacBio sequencing provides useful information supporting early treatment (and adherence counseling) to avoid this outcome [23,24].

#### Case study 2: The PacBio quasispecies-level analysis is able to discriminate different HDRM patterns in the HIV population of a patient

**Figure 5:**
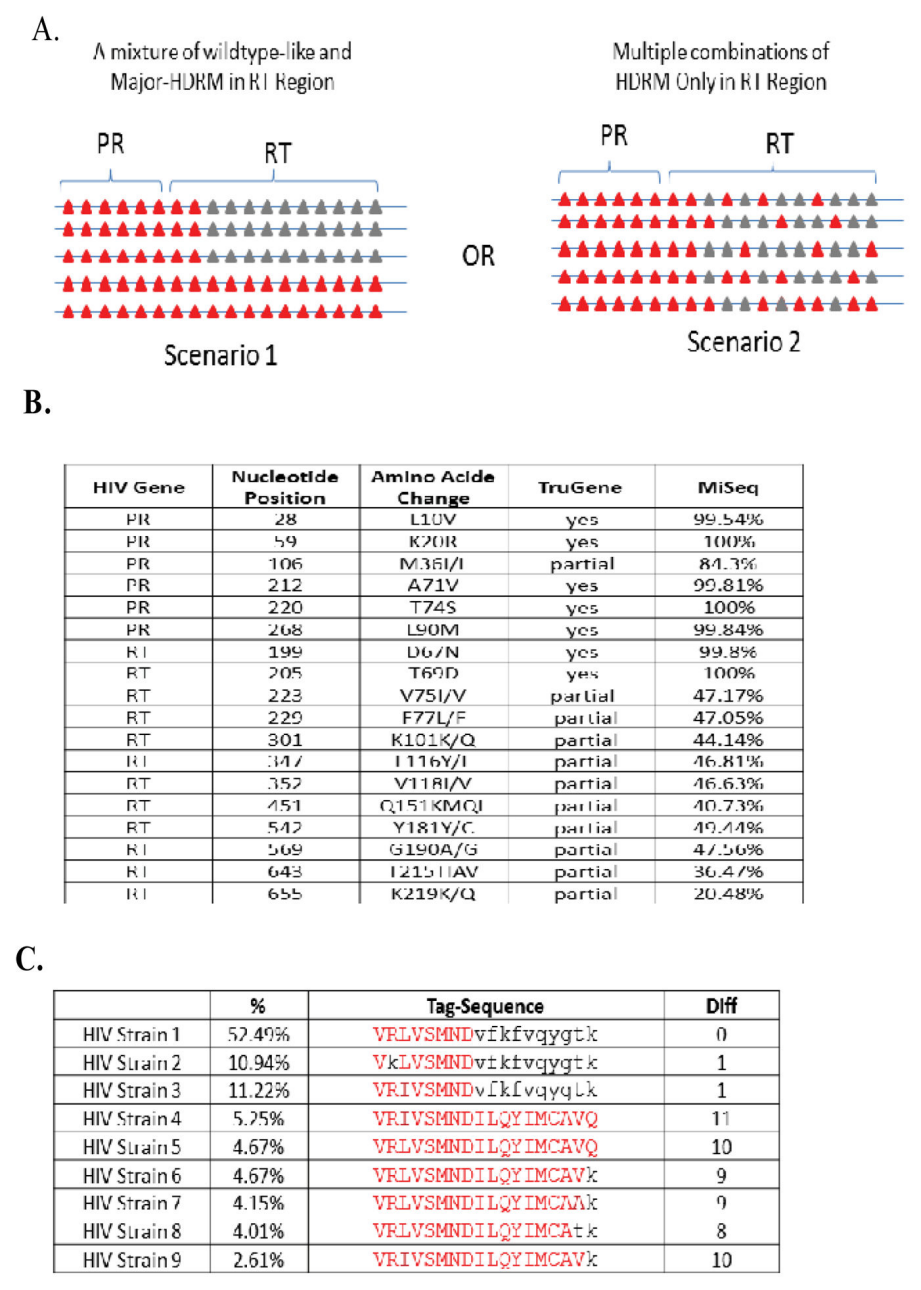
Case study 2 shows the distinct HIV drug resistance mutation (HDRM) profiles associated with different quasispecies A. The hypothesis of two scenarios how the HDRM could be distributed in the HIV quasispecies. The marks in red are denoted as HDRM, and ones in black are wildtype. B. MiSeq and TruGene’s SNV-level analyses show 10 out of 18 HDRMs are co-exist with wildtype sequences on the RT region. The individual SNV-level analysis without linkage information of the HDRM could not provide further detailed understanding how the HDRM distributed across quasispecies as scenario 1 or 2. **C**. PacBio’s quasispecies analysis gives much clearer HDRM profiles at quasispecies level. The results support scenario 1 as demonstrated in A.

A HIV-infected patient had a high HIV viral load of approximately 80,000 copies/ml that was associated with non-adherence to therapy. The TruGene standard drug resistance detection showed that there were six dominant HDRM in PR and two dominant HDRM in RT. In addition, there were10 instances of mixtures of wildtype/HDRM in the RT region (Figure 5A). The question is raised regarding how these mixtures occur at the quasispecies level in the HIV population of the patient. Different answers could potentially cause different treatment approaches. There are two possible scenarios that would result in the detection of mixtures at the SNV level:

1. All of the HDRM are localized to only one quasispecies, thus drugs directed to the RT region may suppress the co-existing wild-type-like virus (Figure 5A).
2. The HDRMs in the RT region are produced by a cumulative effect from different quasispecies, thus anti-RT drugs might be less effective for this patient due to the presence of quasispecies containing some combination of HDRM and an insignificant wild-type-like population (Figure 5A).

Since the TruGene SNV report has neither haplotype information nor quantitative information, the question asked above could not be clarified further (Figure 5B).

To take advantage of MiSeq’s quantitative ability in SNV detection, the same HIV samples were also sequenced by the Illumina MiSeq. MiSeq deep sequencing gave quantitative percentage information for each SNV. Each drug-resistance mutation in the mixtures was present at a frequency of ~ 45% (Figure 5B). Because the HDRMs in the RT region were present in highly similar frequencies, the assumption might be made that the HDRMs might come from the same quasispecies. However, without the direct haplotype information of the HDRM, it is difficult to confidently come to that conclusion.

The same HIV samples were thereafter sequenced by the PacBio system. PacBio was able to generate approximately 20,000 qualified CCS reads in full length or nearly full length. The positions of 18 previously known drug resistance mutations were selected into the tag panel based on the relevancy of the SNVs at locations known to convey drug resistance. The tag-based quasispecies analysis showed that the HDRMs composed approximately 20% of the HIV quasispecies (Figure 5C). In contrast, the remaining 80% of quasispecies had no HDRM in their RT region, i.e., was wildtype with regard to drug resistance. At this point, PacBio quasispecies analysis clearly answered the earlier HDRM question posed regarding the distribution of the mutations among the extant quasispecies. This observation is best explained by the first scenario discussed earlier (Figure 5A). It indicates that anti-RT drugs might still be effective to a certain degree by suppressing the wild-type-like species thereby providing health benefits for the patient.

## Conclusion

The Third Generation Sequencer (TGS), PacBio RS, offers the unique ability of single-molecule, long reads. Combined with the appropriate bioinformatics analysis strategy, PacBio’s long reads can reliably provide HIV quasispecies-level analysis of HDRM with precision as a complement to the traditional SNV-level analysis. The important quasispecies analysis ability of PacBio is very useful in the drug resistance field, and may provide utility for other studies such as receptor tropism, virus evolution, immune evasion, and virion-to-provirus transition.

## Figures and Tables

**Table 1:** Benchmark samples were admixed by BH10 and pNL4-3 strains at serial ratios.

#	Benchmark Sample*	Admixture Ratio*	PacBio CCS Reads**
1	WT BH10	100%	22 K
2	WT pNL4-3 [25]	100%	20 K
3	Admixture 1	10%	18 K
4	Admixture 2	5%	21 K
5	Admixture 3	2.5%	14 K
6	Admixture 4	1.25%	18 K
7	Admixture 5	0.625%	22 k
8	Admixture 6	0.313%	17 k
9	Admixture 7	0.156%	22 k

* The admixture ratios of artificial benchmark samples cover wide range from high to low.

** The corresponding PacBio sequencing typically yields approximately twenty thousands of CCS reads for each sample.
